# Fine-Grained Software Defect Prediction Based on the Method-Call Sequence

**DOI:** 10.1155/2022/4311548

**Published:** 2022-08-03

**Authors:** Fengyu Yang, Yaxuan Huang, Haoming Xu, Peng Xiao, Wei Zheng

**Affiliations:** ^1^College of Aerospace Engineering, Nanjing University of Aeronautics and Astronautics, Nanjing, China; ^2^Software Evaluation Center, Nanchang Hangkong University, Nanchang 330063, Jiangxi, China

## Abstract

Currently, software defect-prediction technology is being extensively researched in the design of metrics. However, the research objects are mainly limited to coarse-grained entities such as classes, files, and packages, and there is a wide range of defects that are difficult to predict in actual situations. To further explore the information between sequences of method calls and to learn the code semantics and syntactic structure between methods, we generated a method-call sequence that retains the code context structure information and the token sequence representing semantic information. We embedded the token sequence into the method-call sequence and encoded it into a fixed-length real-valued vector. We then built a defect-prediction model based on the transformer, which maps the code-vector representation containing the method-call sequences to a low-dimensional vector space to generate semantic features and syntactic structure features and also predicts the defect density of the method-call sequence. We conducted experiments on 10 open-source projects using the ELFF dataset. The experimental results show that the method-call sequence-level prediction effect is better than the class-level effect, and the prediction results are more stable than those of the method level. The mean absolute error (MAE) value of our approach was 8% lower than that of the other deep-learning methods.

## 1. Introduction

The software defect-prediction process involves the extraction of metrics and the construction of a defect-prediction model. In recent years, most traditional software defect-prediction methods use machine-learning algorithms to build defect-prediction models, and the extracted metrics are used as model features [1–3]. Several traditional metrics were devised by researchers to distinguish between defective and nondefective files. However, the traditional metrics mainly focus on code complexity and often neither distinguish programs with different semantics nor fully capture the complex semantic information in the source code and the relationship between code modules. Deep learning can extract features and combine them for higher-level abstraction and learn the essential features hidden in changing data by discovering distributed data representations. Some researchers have used deep learning to automatically capture the semantic representation and syntactic structure of a program.

According to the size of the area in which they appear, defects are divided into two categories: coarse grained (packages, files, classes, etc.) and fine grained (methods, code lines, changes, etc.). Currently, most studies on software defect prediction are based on program modules. However, during program-module integration, specific defects often appear, but only when executing certain instructions, resulting in predictions missing this information. The method invocation level is between the class and method levels and is part of the integration process. Therefore, this study investigates defect prediction from the perspective of method-call sequences. Analyzing the dependencies of methods in the invocation process can obtain contextual information between methods, and this information helps to discover defects arising from the method invocation process. With the impressive results of deep learning models in natural language processing research, researchers have started to use deep learning models to automatically learn semantic features in programs, thus improving the performance of defect prediction. Inspired by this research trend, this study uses a deep learning model to learn the semantic features of a method-call sequence and uses that semantic feature to predict the probability of defects in the method-call sequence.

For the effective use of code semantic information and the potential association between code modules for software defect prediction, we propose a method of fine-grained software defect prediction via the method-call sequence. In the granularity method, we use the transformer to build a defect-prediction model that generates semantic and syntactic structure features (TSASS). It obtains the defect density of the new method-call sequence.

Our study makes the following contributions:

1. We propose a method calling sequence-level defect prediction and construct the defect prediction model TSASS, which uses the transformer model to automatically learn the semantic features of the method calling sequence to construct the defect prediction model and to predict the probability of defects in the method calling sequence.
2. Experiments are conducted on the ELFF dataset, and the results show that our method can effectively improve the prediction performance of the defect prediction model and has the lowest error in terms of comparison with other methods.

The remainder of this paper is organised as follows: Section 2 introduces related work on traditional metric defect prediction, deep-learning metric defect prediction, and fine-grained defect prediction.  Section 3 describes the proposed TSASS approach.  Section 4 presents the experimental setup and parameter settings.  Section 5 analyses the experimental results.  Section 6 provides further exploration and threats to validity.  Section 7 summarises our work.

## 2. Related Work

### 2.1. Defect Prediction Based on Traditional Metrics

Traditional code metrics primarily focus on the statistical characteristics of programs and assume that defective and nondefective modules have distinguishable statistical and object-oriented characteristics. Common traditional metrics are the LOC [3], Halstead metric [4], McCabe metric [5], CK metric [6], MOOD metric [7], and code smell metric [8]. Aman et al. [9] proved that comment lines can also be used as metrics for analysing module defects. Considering that redundant metric elements increase the model construction time, Chen et al. [10] regarded feature selection as a multiobjective problem: one objective is to minimize the number of features selected and the other is to maximize the defect-prediction performance.

### 2.2. Defect Prediction Based on Deep-Learning Metrics

Through observation of the actualised program modules, researchers [11] have found that, sometimes, traditional metrics cannot accurately distinguish if the modules are defective. This is because defective and nondefective modules have the same traditional metrics, thus negatively affecting the training of the defect-prediction model. This phenomenon is caused by the differences in the semantic information and syntactic structure of the modules. It is necessary to establish a more accurate prediction model to distinguish modules with different semantics. At present, researchers [12–15] improve the performance of defect prediction by learning the semantic features of the source code.

Wang et al. first proposed the use of deep learning to automatically capture semantic features. They parsed the source code into an AST and then used a deep-belief network (DBN) to extract token vectors from the AST nodes and generate semantic features. Subsequently, they extracted semantic features from source code changes and extended change-level defect prediction [16]. Deng et al. [17] proposed a defect-prediction framework based on bidirectional long short-term memory network (Bi-LSTM). The long-distance dependence of Bi-LSTM can better learn contextual semantic features in long-sequence data. Dam et al. [18] believed that traversing AST will cause part of the semantic information to be lost and would not reflect the code syntax structure. They proposed a tree-based LSTM (TB-LSTM) method that uses a tree-LSTM to match the AST representation. Shi et al. [19] suggested that adjacent source codes constitute a strong semantic correlation. They used short paths to describe AST terminal nodes and control logic using paired short paths to describe code semantic information.

### 2.3. Defect Prediction Based on Fine-Grained Analysis

Fine-grained analysis is a challenge in the field of software defect-prediction research. Compared with coarse-grained prediction, fine-grained methods can render software testing more reasonable when allocating resources. Recent studies in Java software have shown that the file-level prediction model is more effective than that of the package-level [17–22], and the method-level [23–25] prediction model is more effective than those of the package-level and file-level. Pascarella et al. [25] used different systems and periods to replicate the research of Giger et al. [26] on method-level bug prediction, and they analysed the defect-prediction performance under actual conditions. It is difficult to collect method-level metrics manually. In contrast, deep learning can automatically learn code semantics to easily extract method-level semantic features. Shippey et al. [27] used an AST n-gram to identify defective code features and verified that the AST n-gram has a strong correlation with defects in some systems. Mo et al. [28] proposed a series of metrics in method-level defect prediction and analysed the impact of different metrics on model prediction performance.

We propose a software defect prediction method based on an attention mechanism. The method inputs the vector representation of the code into the TSASS model so as to extract the semantic features and syntactic structure features of the code, which can localize defects to the method-call sequence and finally perform defect prediction to predict the defect density of the method-call sequence and reduce the development cost of developers [29].

## 3. Approach

**Figure 1:**
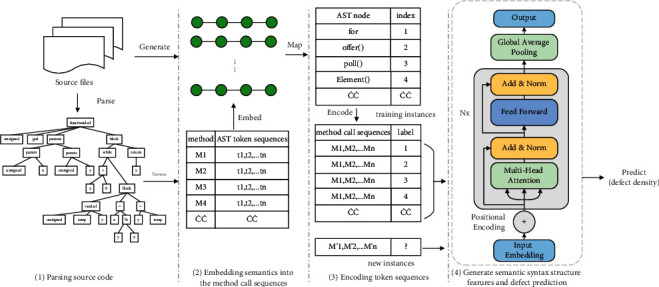
Overview of the proposed approach.

In this section, we introduce in detail the overall architecture of TSASS, which automatically extracts code semantic and syntactic structural features from the source code for defect prediction. As shown in Figure 1, TSASS is divided into four stages. In the first stage, the method-call sequence is extracted from the source code. In the second stage, each method in the source code is parsed into an AST, which is traversed to obtain the token sequence of each method. The token sequence is then embedded in the method-call sequence. In the third stage, one-hot encoding is used to map the token sequence containing the method-call sequence to the numerical space to obtain the token real-valued vector sequence. This contains code semantic information and the syntactic structure. In the fourth stage, the semantic and syntactic structure features are extracted through the TSASS model, and finally, the feature is input into the defect-prediction model for training and prediction.

### 3.1. Parsing Source Code

To obtain the semantic information of each method, we split each Java file into separate method code blocks, parse each method into an AST (the root node of each tree is the method name of the method), and traverse each AST to extract key information nodes. We used the open-source Python package javalang (https://github.com/c2nes/javalang) to parse the source code. First, each class was parsed into an AST. Then, the method declaration node in the AST was traversed to determine the starting line of the first method and the ending line number of the last method in the class file. Finally, the content between the closing brace and the next closing brace was cut into a single method and this was repeated until the end line number was cut. After obtaining a separate method code block, we used javalang to parse each method to obtain a method-level AST.

**Figure 2:**
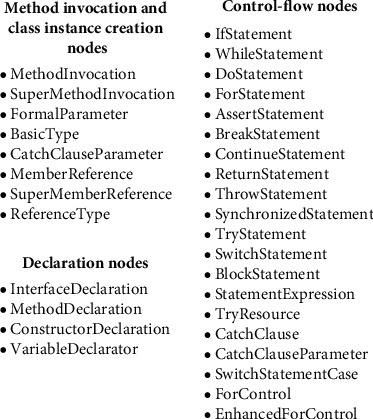
AST nodes.

Several types of AST nodes were identified after parsing. Because some node types had insufficient information and others were too infrequent, we excluded them and only selected three types of nodes on the AST: method invocation and class instance creation nodes, declaration nodes, and control-flow nodes. Because AST is method level, the class declaration node is not extracted. We, therefore, record the method invocation and class instance creation nodes as plain text and mark the method-call structure in parentheses. We then record the node name of the declared node and the node type of the control-flow node.  Figure 2 shows all the selected AST nodes. Algorithm 1 describes the process of parsing the source code.

### 3.2. Embedding Semantics into the Method-Call Sequences

Fine-grained program modules can narrow the range of predicted defects. This method can be developed as a finer-grained program module using Java. The existing method-level defect prediction considers whether the defects are present separately in each method, but the dependence between the methods is relatively strong. The method-call sequence reflects the “interaction” relationship between the different methods, and some defects occur only after a particular method-call sequence. Therefore, we generate the method-call sequence from the project, embed the semantic information extracted from the AST into the method-call sequence, supplement the code hierarchical structure information, and finally encode the sequence into a real-valued vector that can be input to the deep-learning model.

We used the Java ASM (Java bytecode manipulation framework, https://asm.ow2.io/) to extract method-level call relationships to obtain the method-call graph and then used the random-walk technique to traverse the graph to obtain the method-call sequence. Random walk works as follows: starting from a random node, select the next hop from the child node set of the node according to the probability of the walk and repeat the operation until the child node of the current node is empty. At this point, we have reached the end of the call chain. We store the result in a list and repeat it for each current node until we have traversed all the child nodes. The initial node is the head node of the call chain, and the probability of setting the walk is determined by the child nodes. The calculation formula is as follows:

(1) $$\begin{array}{c} \text{seed}=N\%n, \end{array}$$

where *n* is the number of child nodes, *N* is a random number, and the range is *N* ∈ (2*n*, 10*n*).

After obtaining the method-call sequence, we must embed the token sequence formed by parsing the source code into the method-call sequence. According to the method contained in each method-call sequence, the method-call sequence is matched with the corresponding token sequence of the method to obtain the token sequence of the method-call chain. Because the input to the TSASS model is a method-call sequence, the defect density of each method-call sequence must be calculated according to the label of each method to obtain the label of the method-call sequence defined as follows:

(2) $$\begin{array}{c} \text{L}=\sum_{i=1}^{n}\frac{l_{i}}{n}, \end{array}$$

where *l*_*i*_ is the tag value of each method and *n* is the length of the method-call sequence.

### 3.3. Encoding Token Sequences and Handing Imbalance

#### 3.3.1. Encoding Token Sequences

The token sequence obtained by the semantic embedding method-call sequence is a string that cannot be directly input into the deep-learning model. The token sequence must be mapped to the numerical space to obtain a real-valued vector. We used the tokeniser in the Keras (https://keras.io/) library to map the words in the text to the real number vector. First, a mapping dictionary was established between the integers and tokens. Assuming that the length of the token sequence is *n*, each token corresponds to a unique integer, and the mapping range is 1 to  *n*. Then, we calculated the frequency of each token and sorted it according to the token frequency and set the maximum number of words to be retained as num_words_. This ensures that the most common and most frequent num_words_ words are returned. From this, we built an index-mapping dictionary of ordered tokens, with more frequent tokens in the front. Finally, each token is represented as a high-dimensional vector using a dictionary. We set num_words_=2000.

#### 3.3.2. Handling Imbalance

Because the software defect-prediction samples are usually unbalanced (i.e., the samples with defects account for a small part of all samples), if such samples are directly input into the model for training, the prediction results will be biased toward nondefective samples; therefore, the training set must be processed with a data imbalance. Oversampling randomly selects samples from the minority class to replicate, whereas undersampling randomly deletes samples from the majority class until all the classes have the same number of samples. Undersampling may therefore discard some useful data in the dataset [30]. Therefore, to avoid overfitting, we integrate the oversampling and undersampling methods. First, we use random oversampling to copy samples from the minority class, reduce the ratio of defective-to-non-defective samples, and then use random undersampling to delete most samples, adjusting the ratio to 1 : 1 to generate a balanced training set.

### 3.4. TSASS Model

**Figure 3:**
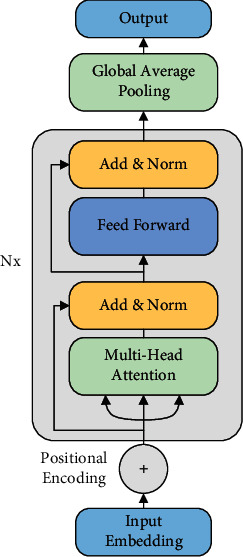
Architecture of TSASS.

Considering that the sequence is relatively long and contains context relations of different lengths, we built a neural network model, called TSASS, based on the transformer. This model comprises an input layer, an encode layer, a global average pooling (GAP) layer, and an output layer. The structure is shown in Figure 3. *N*_*x*_ represents the number of superimposed encode layers.

#### 3.4.1. Input Layer

The input layer is responsible for processing the input of the data. The sequence is first encoded, as stated above, to obtain a real-valued vector. However, the results representing the words are sparse, each word is independent, and the similarity between different words cannot be identified. Because the context between the codes is highly crucial, we use word embeddings to map the features of words to convert them to lower dimensions and create words with the same meaning and similar representations.

Because the length of the sequence is variable and TSASS requires that each token vector input has the same fixed length, we pad the sequence. To avoid overly sparse vectors, the appropriate vector length is selected to add zeros or delete the sequence. After a uniform sequence length, the shorter sequence is filled with several zeros. To prevent the attention mechanism from focusing on the zero-padded data, the sequence must be masked. Certain values must therefore be masked such that they have no effect when the parameters are updated. The specific operation, in this case, is to add an extremely large negative number to the value of these positions and SoftMax, to ensure that the weight of these positions is close to zero.

#### 3.4.2. Encode Layer

The transformer comprises a superposition of the encoder and decoder. The encoder maps the input sequences to continuous representation sequences, and the decoder generates output sequences word by word according to the sequences generated by the encoder. The encoder can extract semantic features and syntactic structure features from the source code; that is, the token sequence is mapped to a continuous representation sequence. Therefore, we use the transformer encoder as the encode layer of the TSASS.

**Figure 4:**
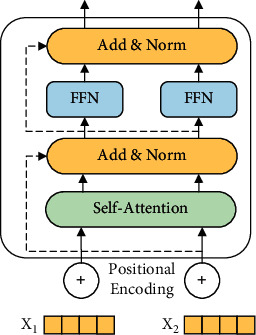
Encode layer.

As shown in Figure 4, the coding layer is composed of multiple self-attention layers, add and norm layer, and feedforward neural network (FFN). The multihead attention layer comprises multiple self-attention layers running in parallel. The position of the token is also an extremely important piece of information in distinguishing the different effects of the defects represented by the method-call sequence in a different location. Because the transformer does not consider the positional relationship of the sequence before inputting the encode layer, the token vector sequence must be positionally encoded. First, a matrix PE that has the same dimension as the input token sequence is constructed, and then it is added to the token vector sequence to obtain the input of the encode layer. The position vector represents the position of each word or the distance between different words, providing effective distance information in the calculation of the multihead attention layer. The PE construction formula is as follows:

(3) $$\begin{array}{c} PE_{\left(\text{pos},\;2i\right)}=\sin\left(\frac{\text{pos}}{10000^{2i/d_{\text{model}}}}\right),\\ PE_{\left(\text{pos},\;2i+i\right)}=\cos\left(\frac{\text{pos}}{10000^{2i/d_{\text{model}}}}\right), \end{array}$$

where pos denotes the position of the current word in the sentence, *i* denotes the ordinal number of each value in the vector, the word embedding value on the even-numbered column is activated by the sine function, and the word embedding value on the odd-numbered column is activated by the cosine function.

The add and norm layer is located around other sublayers to prevent model overfitting, and the gradient disappears during the training process. The add function aims to temporarily remove the neural network units from the network according to a certain probability calculated during the TSASS training process to weaken their dependence on one another. After the network layer, the data are no longer normalised, and the deviation becomes increasingly larger. Therefore, the data must be renormalised to solve the problem of gradient disappearance during backpropagation.

#### 3.4.3. Global Average Pooling Layer

The local features are captured by the encode layer. A fully connected layer is required to integrate the local features through the weight matrix. We built a GAP layer to map the feature map into a vector and perform multiplication operations to achieve dimension reduction. GAP is mainly used to perform a mean pooling of the global features on the features of the last layer to form a feature point, and these feature points are then combined into the final feature vector. Compared with the fully connected layer, GAP is simpler and more able to convert between global features and the final classification, reducing spatial parameters, thus rendering the model highly robust.

#### 3.4.4. Output Layer

After extracting the features from the semantic information and syntactic structure, the features must be input into the machine-learning model for defect prediction. The commonly used machine-learning model is logistic regression, which is generally used to handle classification problems. However, as the original output of logistic regression is the probability, it is a continuous variable. When logistic regression processes continuous variables, the simple least square method must be used for parameter estimation, and the loss function must be set similar to that in the regression task. The output layer of the TSASS is built with a fully connected layer, and the activation function uses a sigmoid to regress the output of the GAP layer. The output result provides the probability that the method-call sequence contains defects.

#### 3.4.5. Training and Optimisation

In the training stage, we use Adam [31] for optimisation, Smooth L1 as the training loss function, and the warmup strategy to update the learning rate. During the initial stage of training, the model is not stable. A larger learning rate increases the difficulty of convergence and causes overfitting. Therefore, warmup must be initiated with a low learning rate at the beginning of training and then switched to a higher learning rate for the usual attenuation after the loss has dropped to a certain extent.

## 4. Experimental Setup

### 4.1. Research Questions

In this section, we present the design of our experiments to verify the effectiveness of TSASS and discuss the following three questions:

1. RQ1: can the method-call sequence-level defect prediction produce better performance than the class-level defect prediction and method-level defect prediction?
2. RQ2: is the TSASS model better than the latest deep-learning model?

### 4.2. Experimental Datasets

To conduct experimental verification and evaluation, we selected 10 Java open-source projects in the ELFF [32] datasets as our evaluation dataset. The ELFF datasets contain information on 23 Java defects at the class and method levels of the open-source project and have been used in previous software defect-prediction studies [27].  Table 1 lists the basic information of these projects, where the number of method defects is the number of methods containing defects in the project, and the proportion of method defects is the ratio of the number of method defects to the total number of methods.

### 4.3. Evaluation Metrics

In general, the most commonly used evaluation indicators for evaluating a machine-learning regression model are the mean squared error (MSE), root-mean-squared error (RMSE), and mean absolute error (MAE). We therefore used the MSE and MAE to evaluate our proposed model. These two indicators are often used to evaluate defect density in software defect prediction [33–36].

#### 4.3.1. MSE

MSE is the average of the squared difference between the predicted target value and the actual target value in the dataset:

(4) $$\begin{array}{c} \text{MSE}=\frac{1}{n}\sum_{i=1}^{n}{\left(f_{i}-y_{i}\right)}^{2}, \end{array}$$

where *f*_*i*_ is the expected number of defects in the program module, *y*_*i*_ is the corresponding actual value of the defect, and *n* is the number of modules. The greater the difference between the predicted and the actual values, the greater the square of the resulting positive error.

#### 4.3.2. MAE

MAE measures the average magnitude of the error in a set of predictions, representing the difference between the predicted value and the actual value:

(5) $$\begin{array}{c} \text{MAE}=\frac{1}{n}\sum_{i=1}^{n}\left|f_{i}-y_{i}\right|. \end{array}$$

When the difference is small, owing to the square of the error value, MSE provides more weight to the larger difference, which then increases the average error score. MAE does not assign error weights to different types but makes the error score linearly increase with the increase in the error, thereby compensating for the shortcomings of MSE.

### 4.4. Baselines

To study RQ1 and RQ2 and evaluate the effectiveness of our method, we performed three sets of experimental comparisons:

1. *Verification of the Validity of the Method-Call Sequence*. To study the effectiveness of the method-call sequence-level defect prediction, we compared the prediction models of different levels, namely, class level and method level, to verify the effectiveness of the method-call sequence. The label of the dataset is the number of defects contained in each class/method. The semantic information extracted from the program module and the defect-prediction model is the TSASS model proposed in our research. We performed all the comparative experiments using the best parameters to ensure the validity of the experiment.
2. *Verification of the Effectiveness of the TSASS Model*. Currently, the deep-learning models commonly used for software defect prediction are the convolutional neural network (CNN), recurrent neural network (RNN), and LSTM. To verify the effectiveness of the TSASS model, we compared our model with other deep-learning models in terms of software defect-prediction capabilities. We designed two comparative experiments. We input the proposed method-call sequence into the latest Seml [37] and DP-ARNN [38] models to perform experiments on defect prediction. Both Seml and DP-ARNN transform the AST of the program into vectors and then input them into the RNN model with attention mechanism and LSTM model respectively, which both learn the contextual information of the code automatically and achieve better performance in defect prediction. The TSASS model in this study learns the semantic and contextual information of the sequence of method calls, so these two deep models are used as a baseline approach to verify the effectiveness of TSASS in semantic feature extraction. Further, we used the best model parameters in our experiment. To facilitate comparison, the predicted defect tendency of the Seml and DP-ARNN models was changed to the predicted defect density, and the loss function of the comparison model was changed to Smooth L1, which is consistent with our results.
3. *Verification of Effectiveness of the Overall Method*. To verify the effectiveness of the overall method, we compared the Seml and DP-ARNN methods to our method. Seml uses the continuous bag-of-words (CBOW) model to characterise the program AST with vectors followed by LSTM to extract semantic features from the vectors and predict defects. DP-ARNN uses an RNN with an attention mechanism to automatically learn semantic features from the token sequence extracted from the AST and then uses the attention mechanism to assign higher weights to important features, ignore unimportant information, and finally perform defect prediction. Both Seml and DP-ARNN are class-level defect predictions, and the parameters of the model are used with their corresponding best parameters. For the convenience of the comparison, the output was changed to predict the defect density of each class.

## 5. Results

Table 2 presents the defect information of the method-call sequence of the project, calculated as explained in Section 3.2.

### 5.1. RQ1: Can Method-Call Sequence-Level Defect Prediction Outperform Class-Level Defect Prediction and Method-Level Defect Prediction?

Class-level defect prediction differs from method-level and method-call sequence-level defect prediction. Classes are more than just methods. To fairly compare class-level, method-level, and method-call sequence level methods, we disregard everything other than methods and extract the AST, which is the same as the node type extracted at the method-call sequence level. Class-level and method-level samples also have class imbalance problems. First, random oversampling was performed to adjust the ratio of the minority class to the majority class, and then, random under-sampling was performed to achieve a ratio of 1 : 1. For each experimental dataset, a 4 : 1 ratio was used to divide the training and test sets. The model used TSASS. The label of the test sample was compared with that of the model output, and the MAE and MSE were calculated.

The experimental results of the class level and method-call sequence level are presented in Table 3. The lower MAE and MSE values are presented in bold. The table indicates that 8/10 projects in the method-call sequence have better MAE values than those of the class, and 7/10 projects have better MSE values than those of the class. The MAE of the method-call sequence level is 6.3% lower than that of the class level, and the MSE is 2.33% lower, indicating that the defect prediction of the method-call sequence has an advantage over the class-level one.

Table 4 presents the results of defect prediction at the method-level and method-call sequence level. Tables 5 and 6 indicate that the MAE values of the method-call sequence-level prediction are better than those of the method-level one on 2/10 projects, and the MSE values of 7/10 projects are lower than those of the method-level one. The MAE of the method level is 4.44% lower than that of the method-call sequence level, and the MSE of the method-call sequence level is 1.75% lower than that of the method level. Although the method-level MAE value is lower than that of the method-call sequence, the MSE value is not as low as the method-call sequence, indicating that method-level defect prediction is unstable. However, study results have revealed that the method-level defect-prediction model has poor generalisation. When a more practical strategy is used for evaluation, the performances of all the models will fall significantly, and the results are close to those of random classifiers [25]. From an intuitive perspective, it is more difficult to find defects in large program modules. Fine-grained predictions may be more effective than coarse-grained predictions. Because the method-call sequence is a sequence of relationships between methods, the methods in the sequence are cross-class, and the granularity of the method-call sequence is fine, which is between that of the class level and the method level.

**Figure 5:**
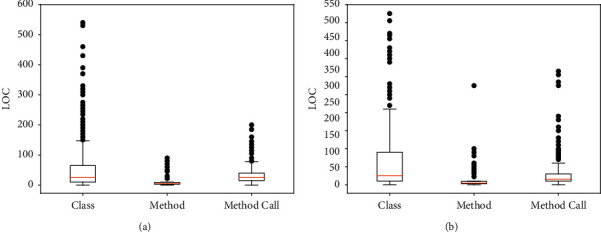
Program module: class, method, and method-call sequence. (a) HTML Unit 2008. (b) Unicore1.4.

We analysed the reasons why method-call sequence is more effective from the perspective of LOC and the number of defects using HTML Unit 2008 and Unicore1.4. Previous studies [39] have used the LOC as a metric.  Figure 5 shows a box plot of the LOC for classes, methods, and method-call sequences. Comparing the median LOCs of the three, in the HTML Unit 2008 project, the median LOC of the class is between 15 and 70, the median of the method-call sequence is between 15 and 40, and the method is between 2 and 12. In Unicore1.4, the median LOCs of the three projects are 10–90, 10–30, and 2–12, respectively. The method-call sequence requires two to three times less work to locate defects than at the class level. If more defects are found in a small number of LOCs, it means that when the same number of LOCs is studied, the method-call sequence can find more defects in quality assurance. Developers can find defects more conveniently based on integration tests. This validates the proposed method-call sequence.

### 5.2. RQ2: Is the TSASS Model Better than the Latest Deep-Learning Model?

We used the Seml and DP-ARNN models for comparative experiments to evaluate the effectiveness of the TSASS model. Seml is a defect-prediction model based on the long- and short-term memory network proposed by Liang et al. [37]. DP-ARNN is a defect-prediction model based on the attention-based recurrent neural network proposed by Fan et al. [38].

We used the same method as the original papers to generate the inputs of Seml and DP-ARNN. When constructing its network structure, we used the same parameter settings as those in references [37, 38].  Table 5 lists the Seml and DP-ARNN inputs for each project. Through comparison and analysis of the MAE and MSE values of the ARNN and TSASS models, 9/10 projects of the TSASS model were found to have lower MAE values than those of Seml, and the values were lower in all the projects than those of DP-ARNN. The MAE value reached 0.095, which is 6% and 21.7% lower than those of Seml and DP-ARNN, respectively. The MSE value was 0.0254, which is 1.23% and 9.36% lower than those of Seml and DP-ARNN, respectively.

**Figure 6:**
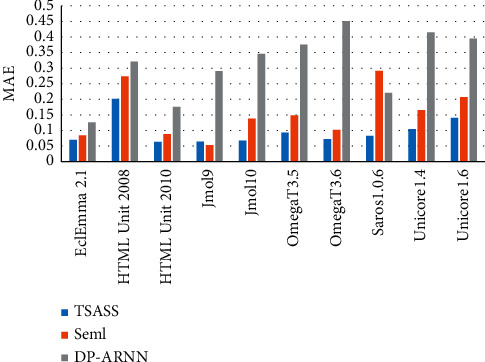
MAE values of different models.

**Figure 7:**
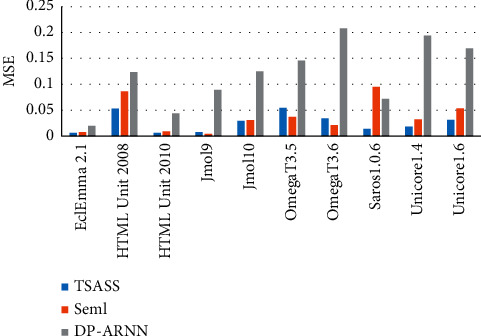
MSE values of different models.

Figures 6 and 7 compare the MAE and MSE values of models. The overall index value of our proposed TSASS model is lower than that of the others, and the model yields better prediction results than those of other models because it not only solves the problem of long-distance dependence of the DP-ARNN model but also solves the parallel problem of the calculation process, so that the semantic syntax structure features extracted by the model can better reflect the code semantics, and the experimental results are more accurate.

**Figure 8:**
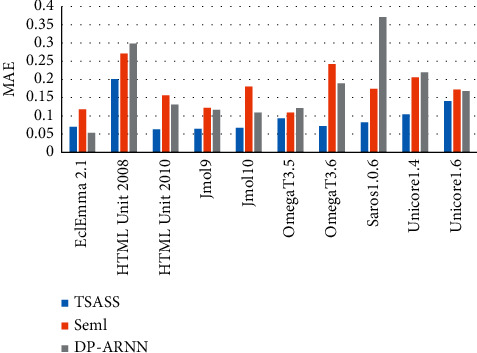
MAE values of different methods.

**Figure 9:**
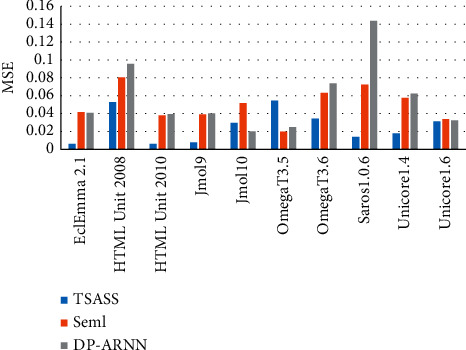
MSE values of different methods.

Figures 8 and 9 compare the MAE and MSE values of the overall methods, respectively. The MAE value of TSASS is generally lower than 0.1, and the MSE value is generally approximately 0.02. The effect of Seml is better than that of DP-ARNN. DP-ARNN is particularly poor for the HTML Unit 2008 and Saros1.0.6 projects.

In summary, our method is superior to class-level defect-prediction and other deep-learning models, and it is better than the Seml and DP-ARNN methods as a whole. We introduce a method-call sequence, which can better express semantic and grammatical structures, according to which the method-call sequence can reduce the size of the program module tested by the developer and can predict specific defects during the integration process.

## 6. Discussion

### 6.1. Further Exploration

Research on software defect prediction in this study has solved some problems, but there is still scope for improvement and research in many areas; therefore, future research methods can focus on the following aspects:

1. Aiming at the characteristics of Java language programs, this paper proposed the use of the method-call sequence to reflect the code hierarchy and predict the defect density of the method-call sequence. Future research can build a semantic and syntactic structure feature extraction model for different programming language characteristics without losing the code structure.
2. This paper constructed a method-call relationship key-value pair collection, which is similar to a tree structure because methods can call each other, and the tree can be treated as a graph structure. This study uses the idea of a random walk to extract the method-call sequence from the program. In graph embedding, there are better random-walk algorithms. In the future, the method-call sequence generation process can be improved.
3. In this study, to obtain semantic information from the source code, the code is converted into an AST, and then, the depth-first traversal method is used to convert it into a sequence. In addition to the AST, control-flow graphs can represent code semantics and syntactic structures. Future research can express the code with a control-flow graph and so forth and then use the graph-embedding technology to learn the semantic structure information in the graph for defect prediction.
4. Code change-level defect prediction is also a concern for researchers. Current research only considers change information. In the future, we can consider combining change information with semantic structure information as a semantic feature of code change.

### 6.2. Threats to Validity

#### 6.2.1. Implementation of Compared Models

We compared Seml and DP-ARNN with our proposed TSASS. As the original implementation of Seml and DP-ARNN has not yet been disclosed, we implemented these models in Python. For fairness, we used a consistent loss function and class imbalance technology. In the training process, to achieve the best results, the learning rate was fine-tuned. The other parameters strictly refer to the original parameters of the article. This may be different from the original implementation of these methods. TensorFlow was used as the deep-learning framework.

#### 6.2.2. Experimental Result Might Not Be Generalisable

All the experimental projects were derived from ELFF datasets composed of Java projects. Aiming at the characteristics of Java language programs, we proposed the use of the method-call sequence to reflect the code hierarchy and predict the defect density of the method-call sequence. This information may be language specific. Therefore, it may not be universal for the characteristics of different programming languages.

#### 6.2.3. MAE and MSE Are Not the Only Suitable Evaluation Indicators

This study selected MAE and MSE as the evaluation indicators of the prediction model, but other indicators can be used for the regression problem of defect prediction such as the magnitude of relative error (MRE).

#### 6.2.4. Parameter Selection

**Figure 10:**
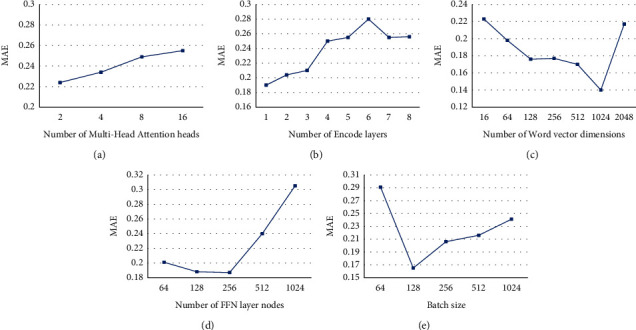
MAE of TSASS under different parameter settings. (a) Multihead attention heads, (b) encode layers, (c) word-vector dimensions, (d) FFN layer nodes, and (e) batch size.

The main hyperparameters that affect the TSASS model are the number of multihead attention heads, the number of encode layers, the number of FFN neurons, the dimension of the word vector, and the batch size. We controlled the other variables to ensure that only one variable changes each time and conducted experiments on the aforementioned parameters to find suitable values. We chose the HTML Unit 2008, Unicore1.4, and Unicore1.6 projects to adjust the parameters and determine the best parameters based on the average of these three projects. As shown in Figure 10, we set the number of multihead attention heads, number of encode layers, number of FFN neurons, dimension of the word vector, and batch size of the MAE value peaks as 2, 1, 1024, 256, and 128, respectively.

## 7. Conclusion

This study proposes software defect prediction at the level of method-call sequences, where method-call sequences can preserve method-to-method dependencies, thus reflecting whether defects will arise during method invocation. In addition, this study constructs a transformer-based deep learning model to automatically learn the semantic information and syntactic structure of method-call sequences. This method can obtain the semantic features in each method and also capture the contextual information between methods during method invocation to find the method-call sequences with high defect risk. This study conducts experiments on 10 open source projects in the ELFF dataset. The results show that defect prediction at the method call sequence level is better than that at the class level and more stable than that at the method level. The TSASS model is also better at learning program semantic features than the deep learning models compared, and the MAE and MSE values of the TSASS model are lower than those of the other deep models. In terms of the overall performance of defect prediction, the MAE and MSE values of the TSASS method are lower than those of the baseline methods.

This study chose to obtain semantic features by analysing the abstract syntax tree of the program when characterizing the method call sequences. In future studies, richer features will be extracted. In addition, future research will be extended to cross-version and cross-project defect prediction.

## Figures and Tables

**Algorithm 1:**
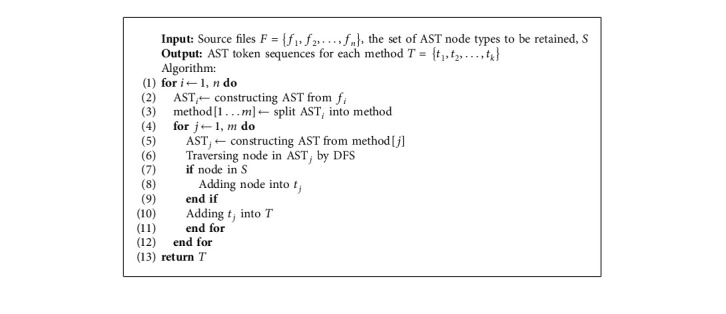
Parsing source code process.

**Table 1:** ELFF datasets.

Project	Methods	Defects	Method defect rate (%)
EclEmma2.1	919	36	3.92
HTML unit 2008	4048	404	9.98
HTML unit 2010	9929	328	3.30
Jmol9	2979	166	5.57
Jmol10	4879	93	1.91
OmegaT3.5	5536	91	1.64
OmegaT3.6	6011	83	1.38
Saros1.0.6	1612	60	3.72
Unicore1.4	2269	176	7.76
Unicore1.6	3937	217	5.51

**Table 2:** Method-call sequence defect information.

Project	Method-call sequence	Defects	Method-call sequence defect rate (%)
EclEmma 2.1	588	20	3.40
HTML Unit 2008	5823	2074	35.62
HTML Unit 2010	1058	73	6.90
Jmol9	1729	168	9.72
Jmol10	3965	356	8.98
OmegaT3.5	6155	338	5.49
OmegaT3.6	7034	302	4.29
Saros1.0.6	1190	102	8.57
Unicore1.4	1103	275	23.11
Unicore1.6	3624	710	19.59

**Table 3:** Comparison of class and method-call sequence.

Project	MAE		MSE	
	Class	Method-call sequence	Class	Method-call sequence
EclEmma2.1	0.157	**0.070**	0.0466	**0.0062**
HTML Unit 2008	**0.160**	0.201	0.0360	**0.0529**
HTML Unit 2010	0.108	**0.063**	0.0314	**0.0062**
Jmol9	0.193	**0.064**	0.0551	**0.0079**
Jmol10	0.338	**0.067**	0.1245	**0.0295**
OmegaT3.5	0.098	**0.093**	**0.0186**	0.0544
OmegaT3.6	0.086	**0.072**	**0.0299**	0.0344
Saros1.0.6	0.255	**0.082**	0.0919	**0.0138**
Unicore1.4	0.134	**0.104**	0.0430	**0.018**
Unicore1.6	**0.056**	0.140	**0.0100**	0.0313
Average	0.158	**0.095**	0.0487	**0.0254**

**Table 4:** Comparison of method and method-call sequence.

Project	MAE		MSE	
	Method	Method-call sequence	Method	Method-call sequence
EclEmma2.1	**0.055**	0.070	0.0211	**0.0062**
HTML Unit 2008	**0.043**	0.201	**0.0383**	0.0529
HTML Unit 2010	**0.029**	0.063	0.0140	**0.0062**
Jmol9	**0.028**	0.064	0.0252	**0.0079**
Jmol10	**0.015**	0.067	**0.0127**	0.0295
OmegaT3.5	**0.014**	0.093	**0.0111**	0.0544
OmegaT3.6	**0.051**	0.072	0.0434	**0.0344**
Saros1.0.6	0.120	**0.082**	0.1158	**0.0138**
Unicore1.4	0.105	**0.104**	0.0898	**0.0180**
Unicore1.6	**0.062**	0.140	0.0583	**0.0313**
Average	**0.051**	0.095	0.0429	**0.0254**

**Table 5:** Comparison of different models.

Project	MAE				MSE					
	Seml	DP-ARNN	TSASS		Seml		DP-ARNN		TSASS	
EclEmma 2.1	0.084	0.126	0.070		0.0076		0.0198		0.0062	
HTML Unit 2008			0.273	0.321		0.201		0.0865	0.1237	0.0529
HTML Unit 2010			0.088	0.176		0.063		0.0091	0.044	0.0062
Jmol9			0.053	0.290		0.064		0.0041	0.0892	0.0079
Jmol10			0.138	0.346		0.067		0.0307	0.1251	0.0295
OmegaT3.5			0.148	0.376		0.093		0.037	0.1456	0.0544
OmegaT3.6			0.102	0.451		0.072		0.0213	0.2079	0.0344
Saros1.0.6			0.291	0.221		0.082		0.0952	0.0721	0.0138
Unicore1.4			0.165	0.415		0.104		0.0324	0.1941	0.0180
Unicore1.6			0.207	0.395		0.140		0.0536	0.1694	0.0313
Average			0.155	0.312		0.095		0.0377	0.1190	0.0254

**Table 6:** Comparison of different methods.

Project	MAE			MSE		
	Seml	DP-ARNN	TSASS	Seml	DP-ARNN	TSASS
EclEmma2.1	0.118	0.053	**0.070**	0.0417	0.0407	**0.0062**
HTML Unit 2008	0.271	0.298	**0.201**	0.0806	0.0955	**0.0529**
HTML Unit 2010	0.156	0.131	**0.063**	0.0380	0.0393	**0.0062**
Jmol9	0.122	0.116	**0.064**	0.0390	0.0402	**0.0079**
Jmol10	0.180	0.109	**0.067**	0.0515	0.0201	**0.0295**
OmegaT3.5	0.109	0.121	**0.093**	**0.0198**	0.0249	0.0544
OmegaT3.6	0.242	0.189	**0.072**	0.0631	0.0739	**0.0344**
Saros1.0.6	0.174	0.371	**0.082**	0.0724	0.1436	**0.0138**
Unicore1.4	0.206	0.219	**0.104**	0.0576	0.0622	**0.0180**
Unicore1.6	0.172	0.168	**0.140**	0.0338	0.0323	**0.0313**
Average	0.175	0.177	**0.095**	0.0497	0.0572	**0.0254**

## Data Availability

The authors selected 10 Java open-source projects in the ELFF [32] datasets as their evaluation dataset. The dataset can be found at “T. Shippey, T. Hall, S. Counsell, et al., So you need more method level datasets for your software defect prediction?: Voila!, Proc. 10th ACM/IEEE International Symposium on Empirical Software Engineering and Measurement. ACM, (2016) 12.”
